# The origin of mechanical harmonic distortion within the organ of Corti in living gerbil cochleae

**DOI:** 10.1038/s42003-021-02540-0

**Published:** 2021-08-25

**Authors:** Wenxuan He, Tianying Ren

**Affiliations:** Oregon Hearing Research Center, Department of Otolaryngology, Oregon Health & Science University, Portland, OR USA

**Keywords:** Cochlea, Hair cell, Transduction, Inner ear

## Abstract

Although auditory harmonic distortion has been demonstrated psychophysically in humans and electrophysiologically in experimental animals, the cellular origin of the mechanical harmonic distortion remains unclear. To demonstrate the outer hair cell-generated harmonics within the organ of Corti, we measured sub-nanometer vibrations of the reticular lamina from the apical ends of the outer hair cells in living gerbil cochleae using a custom-built heterodyne low-coherence interferometer. The harmonics in the reticular lamina vibration are significantly larger and have broader spectra and shorter latencies than those in the basilar membrane vibration. The latency of the second harmonic is significantly greater than that of the fundamental at low stimulus frequencies. These data indicate that the mechanical harmonics are generated by the outer hair cells over a broad cochlear region and propagate from the generation sites to their own best-frequency locations.

## Abstract (web summary)

He and Ren measured sub-nanometer vibrations of the reticular lamina from the apical ends of the outer hair cells in living gerbil cochleae using a custom-built low-coherence interferometer. Their results indicate that mechanical harmonics are generated by the motile outer hair cells and can propagate to their best-frequency locations.

## Introduction

**Fig. 1:**
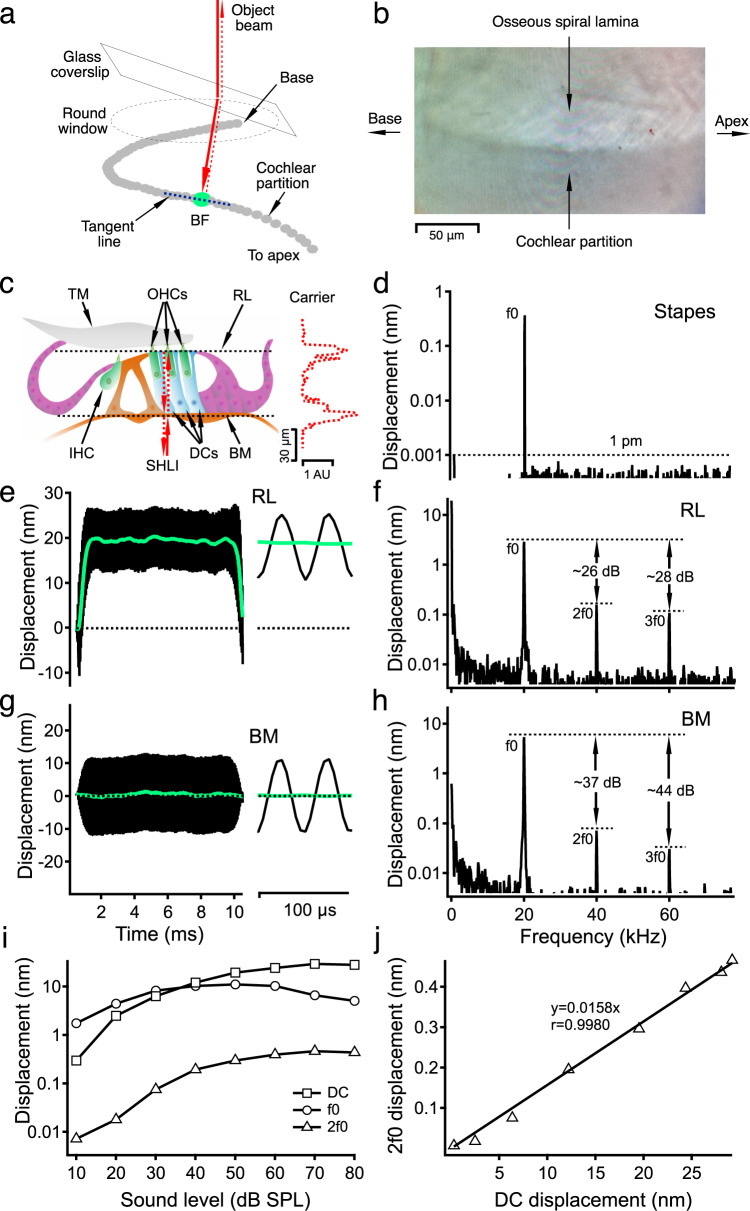
Schematic of optical access to the cochlear partition and vibrations of the reticular lamina and basilar membrane. **a** The object beam of the interferometer is deflected toward the base due to the tilted glass coverslip and refractive index differences among the air, glass, and perilymph. The thick dotted gray line is a mediolateral view of the cochlear partition of the left cochlea in the basal region when the round window is at the top, and the apex is at the bottom. **b** The edge of the osseous spiral lamina is visible over >100-μm distance in the longitudinal direction. **c** Diagram of the organ of Corti. RL reticular lamina, IHC inner-hair cell, OHCs outer-hair cells, TM tectorial membrane, DCs Deiters’ cells, BM basilar membrane, AU arbitrary unit, SHLI scanning heterodyne low-coherence interferometer. The backscattered light level (carrier) as a function of the transverse location (dotted red line) shows two peaks at the reticular lamina and basilar membrane locations. The distance between the two peaks is ~60 μm. **d** The magnitude spectrum of the stapes’ vibration induced by an 80-dB SPL 20-kHz tone. The noise floor is below 1 pm (dotted line), and there is no detectable vibration at 2f0 and 3f0. **e** Displacement of the RL vibration as a function of time (black line) shows an ~20-nm baseline shift toward the scala tympani (solid green line). **f** The magnitude spectrum of the RL vibration shows strong 2f0 and 3f0 harmonics. **g** The time response of the BM shows a symmetrical pattern and no baseline shift. **h** The magnitude spectrum of the BM vibration shows relatively smaller 2f0 and 3f0 than those in the RL vibration. **i** Input–output functions of the reticular lamina DC, f0 and 2f0 displacements. **j** The reticular lamina 2f0 displacement as a function of the DC displacement. The linear regression line with *r* = 0.9980 indicates that 2f0 is closely related to the DC shift. The best frequency is 20 kHz for panels **e**–**h**, and is 21 kHz for panels **i** and **j**.

Speech and music are complex sounds comprising a series of pure tones, in which the frequency of each component tone is an integer (*n*) multiple of the fundamental, the lowest frequency. While the fundamental frequency f0 determines the pitch of the sound, the number, magnitude, and relative phase of harmonics at frequencies 2f0, 3f0, and nf0, define timber or the quality of sounds^1^. When listening to a pure tone, an individual with normal hearing can perceive additional tones at harmonic frequencies as overtones^2^. The ear’s capability to generate and process harmonics has fascinated scientists and musicians for more than a century since the harmonic distortion plays an essential role in speech and music perception. The electrical harmonic distortion has been shown in the activities of the sensory hair cells^3–7^, as well as in auditory nerve responses^8^. Cochlear harmonics have also been found in the basilar membrane vibration and the cochlear fluid pressure in sensitive cochleae^9–15^. The recent micromechanical measurements revealed that the outer-hair cell-driven reticular lamina vibration is more robust and nonlinear than the basilar membrane vibration^16–24^ and that the two tone-induced vibrations in the outer-hair-cell region show rectification and second-order distortions^22^. Although the baseline shift on the basilar membrane at the cochlear base is insignificant in sensitive cochleae^25,26^, except at high sound levels, large baseline shifts have been demonstrated in the tectorial membrane and Hensen’s cell vibrations in the apex^27–29^. These previous observations suggest that the mechanical harmonic distortion may be generated by the outer-hair-cell-based active process at the best-frequency location in the living cochlea. Because the apical ends of outer-hair cells are anchored in the reticular lamina (Fig. 1c), we hypothesize that the single-tone-induced harmonics in the reticular lamina vibration are more robust and have a shorter group delay than those in the basilar membrane vibration. We tested this hypothesis by measuring the reticular lamina and basilar membrane vibrations through the round window (Fig. 1a) at fundamental frequency f0 and harmonic frequencies 2f0 and 3f0 in living gerbil cochleae. The present data demonstrate the single-tone-induced harmonics in the reticular lamina vibration in living cochleae, which indicates that mechanical harmonics are generated by the motile outer-hair cells and can propagate to their best-frequency locations.

## Results

All animals survived anesthesia and surgeries, and the reticular lamina and basilar membrane vibrations showed harmonic distortion products in all sensitive preparations. The data reported below are from ten of eighteen gerbils. The results from other animals are excluded due to the insensitive cochlear conditions at high frequencies, incomplete data sets, or poor signal-to-noise ratio of the vibration measurement.

### Time responses of the reticular lamina and basilar membrane

A representative data set from a sensitive cochlea is presented in Fig. 1e–h. The black lines in Fig. 1e, g are displacements of the reticular lamina and basilar membrane as a function of time. The green lines are low-pass filtered signals of time responses, which indicate the baseline position or DC magnitude. In response to the stapes vibration induced by an 80-dB SPL (0-dB SPL = 20 μPa) tone at 20 kHz (best frequency) (Fig. 1d), the reticular lamina moved toward the positive direction, i.e., toward the scala tympani, and oscillates periodically at the shifted position (Fig. 1e). The magnitude of the baseline shift is approximately 20 nm, indicated by the distance between dotted black lines and solid green lines. The baseline shift was found in the reticular lamina vibration at the best frequency in all sensitive cochleae. The time waveform of the reticular lamina vibration on the right of Fig. 1e shows that the maximal displacement toward the scala tympani (upward) is slightly smaller than that toward the scala vestibuli (downward). In contrast, the overall pattern of the time waveform of the basilar membrane vibration is approximately symmetrical without a clear baseline shift (Fig. 1g).

The magnitude spectrum of the reticular lamina vibration in Fig. 1f shows that the reticular lamina vibrates not only at stimulus frequency f0 but also at harmonic frequencies 2f0 and 3f0. The 2f0 and 3f0 magnitudes are only ~26 to ~28 dB below the f0 magnitude. In contrast, the basilar membrane 2f0 and 3f0 magnitudes in Fig. 1h are more than 37 dB smaller than the f0 magnitude. The zero-frequency peak in the reticular lamina spectrum (Fig. 1f) is approximately 30 dB greater than that in the basilar membrane spectrum (Fig. 1h). The small zero-frequency component in the basilar membrane spectrum likely results from the animal movement because it does not change with the stimulus level or the 2f0 magnitude. The total harmonic distortions (THDs) of the reticular lamina and basilar membrane vibrations were measured at 80-dB SPL when the f0 was equal to the best frequency (BF) in eight sensitive cochleae. The THD of the reticular lamina (4.9±0.7 percent) is significantly larger than that of the basilar membrane (1.4 ± 0.1 percent) (*t* = 4.843, *P* = 0.0010, *n* = 8). Since there is no frequency component at 2f0 and 3f0 in the spectrum of the stapes vibration (Fig. 1d), the data in Fig. 1e–h demonstrate that the observed harmonics in the reticular lamina and basilar membrane vibrations are generated within the cochlea.

The input–output functions of the reticular lamina DC, f0, and 2f0 displacements are presented in Fig. 1i. The data were collected at the 21-kHz best-frequency location from one of the most sensitive cochleae in this study. The f0 increases with sound pressure, peaks at ~40-dB SPL, and then decreases at sound-pressure levels above 50-dB SPL. In contrast, the DC and 2f0 increase with the sound-pressure level with a decreasing rate. While the 2f0 displacement is smaller than the DC displacement, the patterns of 2f0 and DC input–output functions are similar, indicated by the parallel square and triangle lines. The reticular lamina 2f0 displacement is plotted as a function of the DC displacement in Fig. 1j. The linear-regression line and correlation coefficient of 0.9980 indicate that the 2f0 is closely related to the DC displacement.

### The second harmonics in the reticular lamina and basilar membrane vibrations

**Fig. 2:**
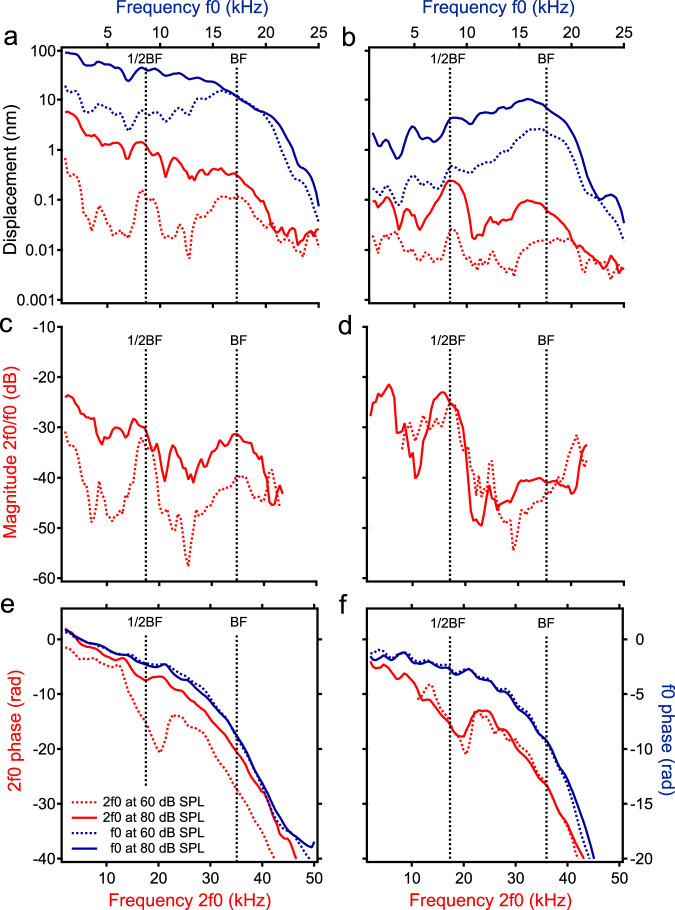
The second harmonics in the reticular lamina and basilar membrane vibrations. **a** Displacements of the reticular lamina (RL) second harmonic (red) and fundamental (blue) as a function of frequency 2f0 (bottom axes) and f0 (top axes) at 60- and 80-dB SPL. While the 2f0 at 60-dB SPL (dotted red line) shows two peaks, one at the best frequency (BF) and the other at 1/2BF, the 2f0 at 80-dB SPL (solid red line) shows a low-pass pattern. The RL f0 shows complete saturation at the BF (blue lines), an indicator of the sensitive cochlear condition. **b** Basilar membrane (BM) 2f0 and f0 responses at 60- and 80-dB SPL. **c** The ratio of the RL 2f0-to-f0 displacement as a function of frequency. **d** The normalized magnitude of the BM 2f0. **e** RL 2f0 (red) and f0 (blue) phase as a function of frequency. **f** BM 2f0 (red) and f0 (blue) phase as a function of frequency. BF: the best frequency.

The magnitude and phase of the second harmonics of the reticular lamina and basilar membrane are presented as a function of frequency 2f0 (bottom axis) in Fig. 2 (red lines). For comparison, the magnitude and phase of the reticular lamina and basilar membrane vibrations at the fundamental frequency f0 (top axis) are also presented (blue lines). While the f0 displacement of the reticular lamina increased proportionally with the sound-pressure level from 60- to 80-dB SPL at frequencies below 10 kHz, it did not increase at frequencies near 17 kHz, the best frequency of the measured cochlear location (blue lines in Fig. 2a). This complete saturation of the reticular lamina vibration indicates the sensitive cochlear condition, under which harmonic distortion products were measured. The displacement of second harmonics of the reticular lamina measured at 60-dB SPL shows two peaks (dotted red line in Fig. 2a), one at the best frequency (~17 kHz) and the other at one-half of this frequency (~8.5 kHz) (referred to the f0 on the top axes). Alternatively, these peaks are at ~34 and ~17 kHz, according to the 2f0 on the bottom axes. The peak centered at the best frequency is broader with a slightly smaller magnitude than that at one-half of the best frequency. As the sound-pressure level increased from 60- to 80-dB SPL, the 2f0 displacement at the best frequency increased at a rate smaller than that at low frequencies, indicated by the uneven space between the dotted and solid red lines. Due to this frequency-dependent increase and the broadening of the peaks, the 2f0 response at 80-dB SPL shows a low-pass pattern (solid red line in Fig. 2a). Compared with the reticular lamina responses, the basilar membrane f0 responses show >10-dB less compressive nonlinearity at the best frequency, indicated by the separated dotted and solid blue lines at ~17 kHz (Fig. 2b). The basilar membrane 2f0 responses show two peaks at ~17 and ~8.5 kHz, not only at 60-dB SPL (dotted red line) but also at 80-dB SPL (solid red line).

The relative magnitudes of the 2f0 are presented by the ratio of the 2f0-to-f0 displacement as a function of frequency in Fig. 2c, d. For a 20-dB increase in the sound-pressure level, the reticular lamina 2f0 increased by ~10 dB, except for that at the one-half of the best frequency (Fig. 2c). At this frequency, the 2f0 displacement at the 60-dB SPL is approximately equal to that at 80-dB SPL. The relative magnitude of the basilar membrane 2f0 hardly changes with the sound-pressure level indicated by the overlapped dotted and solid red lines in Fig. 2d. The level-dependent increase of the reticular lamina relative magnitude (Fig. 2c) indicates that the generation efficiency of the harmonic 2f0 increases with the sound-pressure level.

The reticular lamina and basilar membrane 2f0 (red lines) and f0 (blue lines) phase referred to the stapes vibrations are presented as a function of frequency 2f0 (bottom axes) or f0 (top axes) in Fig. 2e, f. Since the frequency scale of the bottom axis and phase scale of the left axis are twofold of those of the top and right axis, the phase slopes of f0 and 2f0 are comparable in Fig. 2e, f. The reticular lamina and basilar membrane f0 phase decreases with frequency at an accelerated rate, indicating a speed decrease of the traveling wave with frequency. Except for the reticular lamina 2f0 phase at 80-dB SPL, the 2f0-phase patterns (red lines) in Fig. 2e, f are different from those of the f0 phase (blue lines). The 2f0-phase curves include two distinct segments, one at the best frequency and the other approximately at one-half of the best frequency. While the 2f0-phase curves are approximately parallel with the f0-phase curves at the best frequency, their slopes are steeper than those of the f0-phase curves at one-half of the best frequency.

### The third harmonics in the reticular lamina and basilar membrane vibrations

**Fig. 3:**
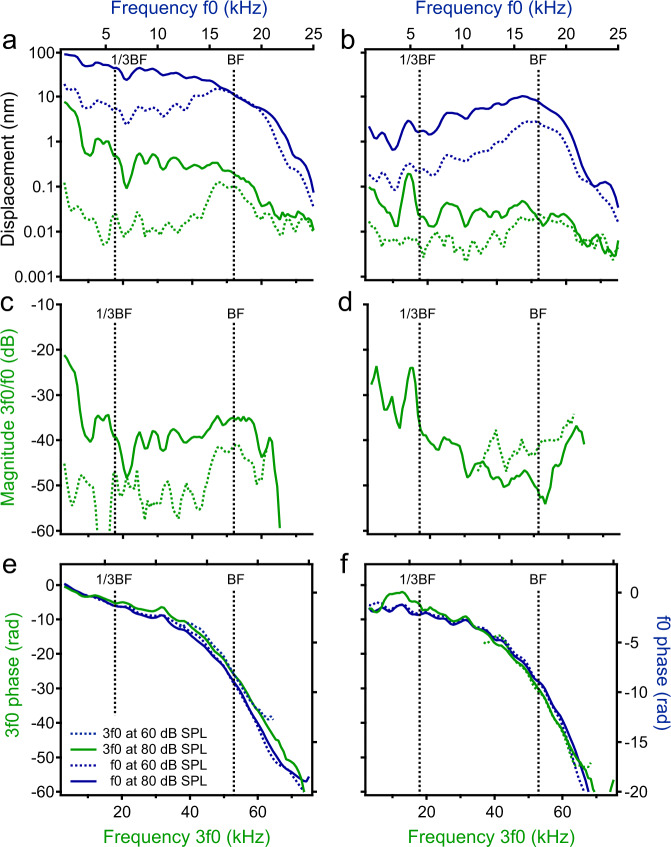
The third harmonics in the reticular lamina and basilar membrane vibrations. **a** Displacements of the reticular lamina (RL) third harmonic (dark green) and fundamental (blue) as a function of the 3f0 (bottom axes) and f0 (top axes) frequency at 60-(dotted lines) and 80-(solid lines) dB SPL. **b** Displacements of the basilar membrane (BM) 3f0 and f0 as a function of frequency. **c** The displacement ratio of the RL 3f0 to f0 as a function of frequency at 60- and 80-dB SPL. **d** The normalized BM 3f0 magnitude at different sound-pressure levels. **e** RL 3f0 (dark green) and f0 (blue) phase as a function of frequency. **f** BM 3f0 (dark green) and f0 (blue) phase. BF: the best frequency.

The data analysis and presentation for Fig. 3 are the same as those for Fig. 2, except for the different harmonic frequency. At 60-dB SPL, the displacement of the reticular lamina 3f0 increases with frequency, reaching the maximum at the best frequency (dotted dark-green line in Fig. 3a). For a 20-dB SPL increase, the 3f0 displacement increased proportionally at frequencies below 30 kHz and only ~6 dB at the best frequency, indicating an ~14-dB compression. Due to this frequency-dependent increase, the 3f0 displacement curve at 80-dB SPL shows a low-pass pattern, without a clear peak at the best frequency (solid dark-green line in Fig. 3a). The displacement of the basilar membrane vibration at frequency 3f0 (Fig. 3b) is smaller than that of the reticular lamina vibration (Fig. 3a) across frequencies. At 60-dB SPL, the 3f0 displacement of the basilar membrane shows a broad peak centered at the best frequency (dotted dark-green line in Fig. 3b). The magnitude of this peak barely increased with the sound-pressure level. While there is no clear peak at the best frequency, a sharp peak at ~6 kHz, approximately one-third of the best frequency, is visible at 80-dB SPL (solid dark-green line in Fig. 3b).

The relative magnitude of the reticular lamina 3f0 at the best frequency is about 40 dB below the f0 at 60-dB SPL (dotted dark-green line in Fig. 3c). It increases with sound-pressure level but at a rate smaller than that of the stimulus, indicated by <20-dB separation between the dotted and solid dark-green line. The relative magnitude of the basilar membrane 3f0 decreases with the sound pressure level at the best frequency (dark-green curves in Fig. 3d). At 80 dB SPL, the peak at about one-third of the best frequency is >25-dB larger than that at the best frequency.

The 3f0-phase responses of the reticular lamina and basilar membrane are similar to those of the f0 (Fig. 3e, f).

### The time relationship between harmonics and fundamental

**Fig. 4:**
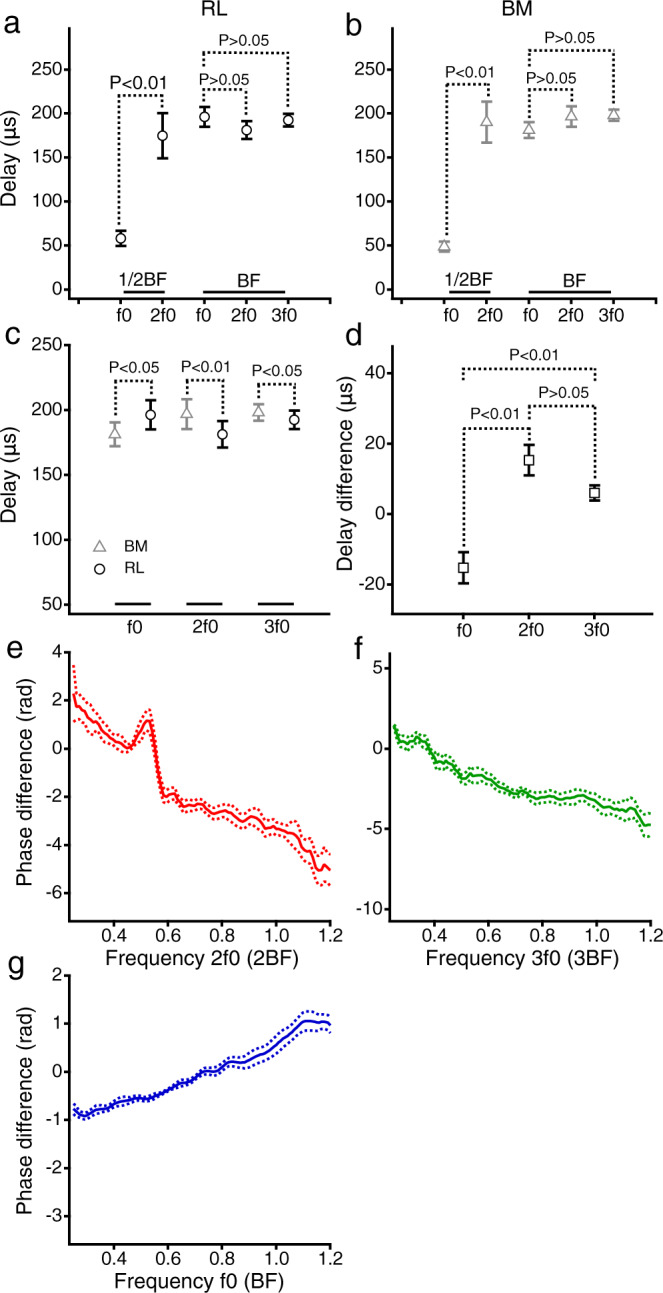
Time relationship between the reticular lamina and basilar membrane vibration at harmonic and fundamental frequencies. **a** The reticular lamina (RL) 2f0 group delay is significantly larger than the f0 group delay at one-half of the best frequency, while there is no significant difference among the f0, 2f0, and 3f0 group delays at the best frequency. **b** The basilar membrane (BM) 2f0 group delay is also greater than the f0 group delay at one-half of the best frequency. **c** The RL 2f0 and 3f0 group delays are significantly smaller than the BM group delays, while the RL f0 group delay is larger than the BM f0 group delay. **d** The 2f0 and 3f0 group-delay differences between the RL and BM are significantly larger than the f0 group delay difference. **e**, **f** The phase differences between the RL and BM 2f0 and 3f0 as a function of frequency. The downward slopes of the phase-difference curves confirm that the RL 2f0 and 3f0 group delays are smaller than those of the BM. **g** The f0 phase difference between the RL and BM vibrations as a function of frequency. The upward phase slope of the f0 phase-difference curve indicates that the f0 arrives at the RL after the BM vibration. The solid and dotted lines in panels **e**–**g** represent the means and standard errors, and *n*=8.

The group delays of the f0, 2f0, and 3f0 were derived from the corresponding phase slopes of the reticular lamina and basilar membrane vibrations at 80-dB SPL, and the means and standard errors of the group delays from ten sensitive cochleae are presented in Fig. 4a, b. A paired t-test shows that the reticular lamina 2f0 group delay (175 ± 25 μs) is significantly greater than the f0 group delay (58 ± 9 μs) at one-half of the best frequency (*t* = 4.9161, *P* = 0.0008, *n* = 10) (Fig. 4a). Similarly, the basilar membrane 2f0 group delay (190 ± 23 μs) is also greater than the f0 group delay (49 ± 6 μs) (*t* = 6.3148, *P* = 0.0001, *n* = 10) when the stimulus frequency is at one-half of the best frequency (Fig. 4b). If the 2f0 was exclusively generated at the measured cochlear location, the 2f0 group delay should be comparable to the f0 group delay. The observed group-delay differences suggest that, when f0 is at one-half of the best frequency, the reticular lamina and basilar membrane 2f0 may not be entirely generated at the measured cochlear location. A one-way ANOVA analysis shows no significant difference among the reticular lamina f0, 2f0, and 3f0 group delays when the f0 equals the best frequency (*F* = 0.6664, *P* = 0.5217) (Fig. 4a). A similar relationship was found among the basilar membrane f0, 2f0, and 3f0 group delays (*F* = 1.0156, *P* = 0.3756) (Fig. 4b). The similar group delays of the f0, 2f0, and 3f0 at the best frequency indicate that the f0 group delay dominates the 2f0 and 3f0 group delays and that the 2f0 and 3f0 are generated locally at the measured cochlear location.

### The time relationship between the reticular lamina and basilar membrane harmonics

The reticular lamina 2f0, 3f0, and f0 group delays are compared with the corresponding basilar membrane group delays in Fig. 4c. The group delays used for the comparison are the same as those in Fig. 4a, b at the best frequency. A paired *t*-test shows that the reticular lamina 2f0 group delay (181 ± 10 μs) is significantly smaller than that of the basilar membrane (197 ± 12 μs) (*t* = 3.5414, *P* = 0.0063, *n* = 10). Moreover, the reticular lamina 3f0 group delay (193±7 μs) is also smaller than that of the basilar membrane (198 ± 6 μs) (*t* = 2.4376, *P* = 0.0375, *n* = 10). In contrast, the reticular lamina f0 group delay (197 ± 11 μs) is significantly larger than that of the basilar membrane (181 ± 9 μs) (*t* = 3.1097, *p* = 0.0125, *n* = 10).

The group-delay differences between the reticular lamina and basilar membrane were quantified by subtracting the reticular lamina group delays from the basilar membrane group delays, and the means and standard errors are presented in Fig. 4d. A one-way ANOVA test reveals significant differences among the f0, 2f0, and 3f0 group-delay differences (*F* = 15.6052, *P* = 0.0001). The multiple comparisons using the Tukey test show that the f0 group-delay difference (−15 ± 5 μs) is significantly smaller than those of the 2f0 (15 ± 4 μs) (*Q* = 7.7240, *P* = 0.0001, *n* = 10) and 3f0 (6 ± 2 μs) (*Q* = 5.0308, *P* = 0.0024, *n* = 10) and that there is no significant difference between the 2f0 and 3f0 group-delay difference (*Q* = 2.4232, *P* = 0.2184, *n* = 10).

Since the vibration phase ($\triangle \varphi$) is determined by the frequency $\left(\triangle f\right)\,$and group delay ($\tau$) $(\tau =-\triangle \varphi /\triangle \omega =-\frac{\triangle \varphi }{2\pi \triangle f})$, a group-delay difference should result in a phase difference between the reticular lamina and basilar membrane vibration. The phase differences were obtained by subtracting the reticular lamina phase from the basilar membrane phase in eight animals. The means and standard errors of the phase differences are presented as a function of the normalized frequency in Fig. 4e–g. The downward slopes of the phase-difference curve in Fig. 4e, f confirm that the reticular lamina 2f0 and 3f0 occur before the basilar membrane harmonics. The upward phase slope in Fig. 4g is consistent with the observation that the basilar membrane vibrates earlier than the reticular lamina at the f0 frequency^17^. The opposite slopes of the harmonics and fundamental suggest that the 2f0 and 3f0 are generated not by the sound source or the middle ear but rather by motile outer-hair cells. The peak of the 2f0 phase-difference curve in Fig. 4e likely results from the phase transition between the propagated and nonpropagated 2f0 components, which are discussed below.

## Discussion

The current heterodyne low-coherence interferometry demonstrates the mechanical harmonic distortion in the reticular lamina vibration in living gerbil cochleae. The reticular lamina harmonics have larger magnitudes (Fig. 1f, Fig. 2a, b, Fig. 3a, b) and smaller group delays (Fig. 4c–f) than the basilar membrane harmonics. The latency of the second harmonic is significantly greater than that of the fundamental when the stimulus frequency is at one-half of the best frequency (Fig. 4a, b). Since the reticular lamina harmonics are measured from the apical ends of the outer-hair cells, the present results indicate that mechanical harmonics are generated by motile outer-hair cells over a broad region along the reticular lamina and then propagate to their best-frequency locations.

**Fig. 5:**
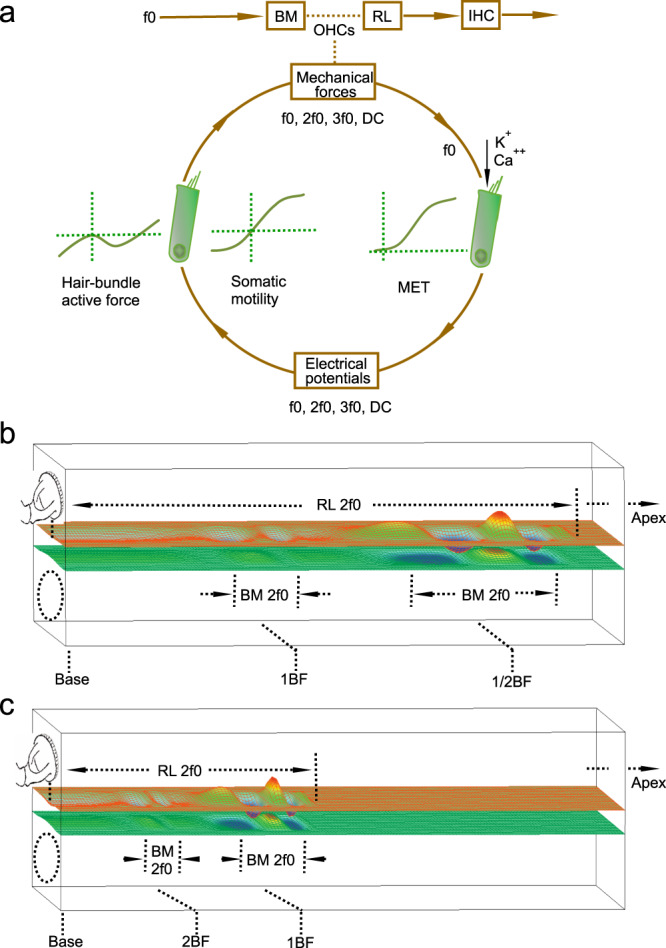
Illustration of the generation and propagation of harmonic distortion products. **a** Diagram of the cochlear active process and the generation of harmonics. In response to the fundamental tone f0, nonlinear mechanoelectrical transduction of the outer-hair cells generates receptor potentials at f0, 2f0, 3f0, and other distortion frequencies. The somatic and hair-bundle motilities generate forces at f0 and harmonic frequencies and result in movements at the apical ends of outer-hair cells, which are measured as the reticular lamina vibrations at fundamental and harmonic frequencies f0, 2f0, and 3f0. BM basilar membrane, RL reticular lamina, IHC inner-hair cell, MET mechanoelectrical transduction, DPs distortion products. **b** When the f0 is one-half of the best frequency of the measured cochlear location, the peak of the f0 vibration is at an apical location indicated by 1/2BF peak. After it is generated by outer-hair cells over a broad region along the cochlear partition, the 2f0 harmonic travels from the generation sites to its own best-frequency location indicated by 1BF peak and is measured as the low-frequency peak in the BM and RL vibrations (Fig. 2a, b). **c** When the stimulus frequency is equal to the best frequency of the measured location, a maximal 2f0 vibration is recorded at the longitudinal location 1BF peak. Upon generation of the 2f0 by outer-hair cells, the 2f0 travels to its best-frequency location 2BF peak. This vibration was not detected in this experiment because it is at a basal location far from the measurement site.

When a tone is delivered to the ear, the air-pressure oscillation in the ear canal causes the eardrum to vibrate. This air-borne vibration is efficiently transmitted into the cochlear fluid through the miniature middle-ear bony chain. The resultant fluid pressure initiates a vibration of the basilar membrane. This vibration travels from the cochlear base to the apex and results in a maximum vibration at a cochlear location determined by the stimulus frequency^30^. Low-frequency vibrations are close to the apex, and high-frequency ones are near the base (Fig. 5b, c). When the basilar membrane moves toward the scala media, the shearing motion between the reticular lamina^19,20,23,24,31,32^ and the tectorial membrane^33^ (Fig. 1c) deflects the hair bundle toward the tallest stereocilia, which opens mechanoelectrical transduction channels by pulling on the tip links^34^. The electrical potential gradient across the cellular membrane drives K^+^ and C_a_^++^ from endolymph into outer-hair cells through mechanoelectrical transduction channels^35–38^. This sound-modulated current results in a change in membrane potential at the f0^39,40^. In response to the membrane potential change, outer-hair cells produce forces at f0 through somatic and hair-bundle motilities^36,41–48^ (Fig. 5a). With appropriate timing, the outer-hair cells from a broad region work collaboratively to boost the vibration at the best-frequency location^17^.

Due to the inherent nonlinearity of the mechanoelectrical transduction^35,36^, in response to a single tone, the outer-hair cells generate receptor potentials not only at the fundamental frequency f0 but also at harmonic frequencies 2f0 and 3f0, and a DC potential^49,50^. These receptor potentials can also result in the length change of outer-hair cells (Fig. 5a). The observed reticular lamina vibration at harmonic frequencies from the apical ends of the outer-hair cells indicates that the outer-hair cells can generate mechanical harmonic distortion in living cochleae^51^. According to the frequency-location map in the gerbil cochlea^52^, the broad spectra of the reticular lamina harmonics (Figs. 2a and 3a) indicate that the outer-hair cells generate harmonics not only at the best-frequency location but also at a broad region basal to this location. This is consistent with recent observations that the robust reticular lamina vibrations at low frequencies are physiologically vulnerable and suppressible^16,17,32^, and the two-tone-induced distortion products in the reticular lamina vibration extended to low frequencies^32^.

In most nonlinear systems, the quadratic nonlinearity results in the DC component and even-order distortion (such as the second harmonic), and the cubic nonlinearity generates odd-order distortion (such as the third harmonic). It was a surprising finding that the harmonic in the basilar membrane vibration was dominated by the second harmonic without substantial DC shift in the sensitive cochleae^11^. The present data in Fig. 1e, f show that both the DC shift and 2f0 coexist in the reticular lamina vibration. Fig. 1i, j shows that DC and 2f0 displacements increase nonlinearly at a similar rate with the sound-pressure level and that the DC displacement is closely related to the 2f0 displacement. These data indicate that, during the stimulation, the outer-hair cells change their length, which can shift the operating point of mechanoelectrical transduction to a more nonlinear range^53,54^. This consequently increases production of the electrical and mechanical harmonics. While the DC displacement larger than f0 displacement (Fig. 1e, f) could be interpreted as a result of the adaptation of the outer-hair-cell mechanoelectrical transduction process^55–58^, the constant DC and f0 displacements during the stimulation and the fast onset and offset of the responses do not support this interpretation.

Since the outer-hair cells are connected directly to the reticular lamina and indirectly to the basilar membrane via Deiters’ cells (Fig. 1c), the outer-hair-cell-generated harmonic force can vibrate the reticular lamina and basilar membrane at the generation sites. The magnitude and phase of the resultant vibrations are determined by the mechanical properties of the cochlear partition. While the flexible reticular lamina allows robust 2f0 responses at a broad frequency range (Fig. 2a, c) and the baseline shift (Fig. 1e), the stiffness gradient of the basilar membrane allows sensitive 2f0 responses only at the best frequency and one-half of the best frequency (Fig. 2b, d).

The cochlea-generated harmonics may travel to their own best-frequency locations as acoustically and electrically evoked otoacoustic emissions^59–70^. When the f0 is at one-half of the best frequency, the harmonics are mainly generated at the cochlear locations apical to the measured location (indicated by 1/2BF peak in Fig. 5b) according to the cochlear frequency-location map^52^. When the resultant 2f0 harmonic travels to its best-frequency location (i.e., the measured location, indicated by 1BF peak in Fig. 5b), it is amplified by outer-hair cells. This amplified propagated 2f0 is measured as the low-frequency peak (Fig. 2a, b) in this experiment. Thus, the latency of the 2f0 peak at one-half of the best frequency includes parts of the f0 and 2f0 traveling-wave delays, and it is significantly larger than that of the f0 (Fig. 4a, b). When the f0 is equal to the best frequency, the harmonics are mainly generated by the outer-hair cells at the measured cochlear location (1BF peak in Fig. 5c), forming the harmonic peaks at the best frequency (red lines in Fig. 2a, b). Although the 2f0 generated at the measurement location may also travel to its own best-frequency location (indicated by 2BF peak in Fig. 5c), this amplified peak is not detectable in this experiment because it is at a basal location far from the measurement site (1BF peak in Fig. 5c).

Therefore, both the local nonpropagated and propagated components contribute to the harmonics in the reticular lamina and basilar membrane vibrations. Each component’s contribution varies with the stimulus frequency, the sound-pressure level, and the cochlear location where the vibration is measured. These factors can contribute to the harmonic group delays in the basilar membrane and reticular lamina vibrations (Fig. 4a, b) due to the group-delay difference between the propagated and nonpropagated components. Consequently, the steep phase slope and long group delay of the harmonics are observed only when the harmonics are dominated by propagated components. Under the current experimental conditions, the single-tone-induced second harmonic is dominated by the propagated component only at one-half of the best frequency (Fig. 2e, f).

The steep 2f0 phase slope and magnitude peak at one-half of the best frequency may also result from a 2f0 forward-traveling wave, which originates at a location basal to the measured place. When the f0 is one-half of the best frequency, the f0 traveling wave peaks at a cochlear location apical to the measurement site, as indicated by 1/2BF in Fig. 5b. The 2f0 can be generated not only at the 1/2BF but also over a broad region basal to this place. The 2f0 component generated at the location basal to the measured place can propagate and be amplified by outer-hair cells like an external tone-induced traveling wave. Although the 2f0 may also be generated locally at the measured location (1BF in Fig. 5b), it may not be amplified without propagation. Thus, the 2f0 generated at the basal region can dominate the vibration and result in the steep-phase slope and the magnitude peak at the measurement location (1BF in Fig. 5b).

Although the detailed propagation mechanisms are subject to further investigation, the 2f0 phase and magnitude data indicate that the harmonics can propagate to their best-frequency location. When the harmonics reach their best-frequency locations, the optimal vibrations can excite the auditory sensory cells and nerve, resulting in the perception of additional tones at harmonic frequencies. This finding is consistent with a recent study (Fig. 2 and supplementary Fig. 1 by Vavakou et al.)^22^, which indicates that second-order distortion products propagate in the organ of Coti.

There is concern that the reported phase difference between the reticular lamina and basilar membrane vibrations may be due to a tangential approach angle of the object beam with the reticular lamina and basilar membrane. Our measurement and calculation based on the cochlear place-frequency map in the gerbil^52^, show that the 20-kHz location is ~2.4–2.5 mm from the base, which is consistent with previous mechanical measurements^71–74^. The best-frequency locations closer to the cochlear base and the use of the glass coverslip with a tilted angle in this study allow the deflected object beam to access the cochlear partition approximately in the perpendicular direction (Fig. 1a). This was indicated by the image through an objective lens with a numerical aperture of 0.28 in which the landmarks of the cochlear partition are visible over a >100-μm distance in the longitudinal direction (Fig. 1b). Under this condition, the measurement error due to the nonperpendicular angle of the object beam is insignificant. Moreover, the following previous studies demonstrate that the phase difference between the reticular lamina and basilar membrane vibrations is not a measurement artifact, instead, it is a manifestation of the cochlear active process. The reticular lamina–basilar membrane phase difference has been found in the mouse and gerbil cochleae^16,17^, despite different measurement angles resulting from anatomical differences. The phase and magnitude differences vanish immediately upon the animal’s death^17^. The time-domain click responses show that the reticular lamina and basilar membrane move in opposite directions at the beginning of the response and in the same direction during the lasting oscillation (Fig. 4 by He et al., 2018)^17^. Since the early part of the click response represents the low-frequency component, and the late part is dominated by the best-frequency component, the click response confirms the frequency-dependent phase difference between the reticular lamina and basilar membrane vibration. Moreover, the small phase difference between two longitudinal locations at low frequencies^71^ may not account for a 180-degree phase difference between the reticular lamina and basilar membrane vibrations.

In summary, present heterodyne low-coherence interferometry demonstrates in vivo that reticular lamina harmonics are significantly larger and have shorter group delays than the basilar membrane harmonics. The latency of the second harmonic is significantly greater than that of the fundamental at low frequencies. These results indicate that harmonics are generated by motile outer-hair cells and propagate to their own best-frequency locations. This knowledge is critical for understanding speech and music perception and may be applied for designing new cochlear prostheses for restoring hearing to patients with hearing loss.

## Methods

### Animals

Eighteen male and female Mongolian gerbils with normal hearing at the age of 4–8 weeks (40–80 g) were used in this study. The animal use protocol was approved by the Oregon Health & Science University Institutional Animal Care and Use Committee (Protocol Number: IP00000932).

### Measurement of the reticular lamina and basilar membrane vibrations

The experiments were conducted on a vibration-isolation table inside an acoustically attenuated and electrically shielded booth. Anesthesia was induced by ketamine and xylazine (100 mg per kg and 10 mg per kg intramuscularly). A tracheotomy was performed to keep natural free breathing. Body temperature was monitored with a rectal temperature probe and kept constant at ~38 °C. The animal’s head was held using a custom-built head holder mounted on a three-dimensional translational stage. The auditory bulla on the left side was exposed through a ventrolateral surgical approach. An acoustic probe consists of two speakers and one microphone was connected to the ear canal to form a closed sound field. Two continuous tones at f1 and f2 frequencies of 20 and 24 kHz and at 60-dB SPL were presented, and the evoked distortion product otoacoustic emission at 2f1–f2 (16 kHz) was displayed on a signal analyzer (SR785, Stanford Research Systems, Sunnyvale, CA) and digitized using a lock-in amplifier (SR830 DSP, Stanford Research Systems, Sunnyvale, CA). Cochleae with a <5-dB decrease of the otoacoustic emission were considered a sensitive preparation. The sensitive cochlear condition was confirmed by the nonlinear compression of the reticular lamina and basilar membrane vibrations.

The anterior and lateral bony walls of the bulla were carefully removed using a sharp blade to visualize the round window^59,60,74^. After the round-window membrane was removed with a tungsten hook, the animal’s head was turned to a position with the round window at the top and the apex at the bottom. One end of a glass-coverslip strip with ~1.5-mm width and 0.17-mm thickness was placed on the posterolateral bony edge of the round window and the other end on the tympanic ring and the bony edge of the bulla (Fig. 1a). It usually took a few minutes for perilymph to fill the space between the glass coverslip and the round window. Since the edge of the tympanic ring is in a higher plane than the round window, an angle was formed between the glass coverslip and the horizontal plane. This angle was adjusted by lifting or lowering the glass coverslip at the tympanic-ring end. Due to refractive-index differences among the air, glass, and perilymph, the glass coverslip and the perilymph below deflected the object beam toward the cochlear base and brought it close to the perpendicular direction to the cochlear partition (Supplementary Notes). A light beam through a single-mode optical fiber was brought close to the lateral bony wall of the scala vestibuli to illuminate the cochlear partition. The position and orientation of the optical fiber were adjusted so that landmarks of the cochlear partition were visible. When the object beam was approximately perpendicular to the tangent line of the cochlear partition at the best-frequency location, the low-coherence light from the object arm of the interferometer^16,75^ was focused on the center of the outer-hair-cell region through a long working-distance objective lens (Plan Apo 20X, NA 0.28, Mitutoyo, Japan). The cochlear-partition position was confirmed by the video image through the objective lens, in which the edge of the osseous spiral lamina is clearly visible over >100-μm distance in the longitudinal direction (Fig. 1b). The high sensitivity, wide dynamic range, and low phase noise make this interferometer suitable for measuring low-level harmonic distortion in reticular lamina vibration. After the transverse locations of the basilar membrane and reticular lamina were determined by the peaks of the carrier signal^17^ (Fig. 1c), the object light beam of the interferometer was focused on those locations sequentially for vibration measurements.

### Signal generation and data acquisition

A sinusoidal signal at frequency f0 with durations of 20 or 10 ms and 1-ms onset and offset were generated by a digital-to-analog converter of a dynamic signal analyzer (PXI-4461, National Instruments, Austin, TX) at a sampling rate of 200,000 samples per second. The signal analyzer was controlled by a controller (PXIe-8105, National Instruments, Austin, TX) and custom LabVIEW-based software. The signal from the digital-to-analog converter was used to drive an electrostatic speaker (EC1, Tucker–Davis Technologies, Alachua, FL) through a power amplifier. The sound-pressure level near the tympanic membrane was measured using a probe microphone and controlled by changing the signal level to the power amplifier. For determining the best frequency of the measured cochlear location, the magnitude and phase of the reticular lamina and basilar membrane vibrations were measured at different frequencies and sound-pressure levels. The best frequency was determined by the frequency with the maximum magnitude of the basilar membrane response to 40-dB SPL tones.

The output voltage from a displacement decoder (DD-500, Polytec Inc., Irvine, CA) of the interferometer was digitized by the analog-to-digital converter of the same signal analyzer at the same sampling rate as for signal generation. Acquired signals were averaged 20–50 times, depending on the signal-to-noise ratio. Data with animal respiration- and heartbeat-induced artifacts were identified and discarded during the averaging^17,32^. Spectra of the averaged signals were obtained through fast Fourier transforms, and RMS (root mean square) magnitude and phase of the displacement at fundamental frequency f0 and harmonic frequencies 2f0 and 3f0 were obtained. The data were collected at sound levels 40-, 60-, and 80-dB SPL and at frequencies from 0.5 kHz to 30 kHz. For reliably measuring the phase patterns of the harmonics, a small frequency step of 250 Hz was used in this experiment. To ensure that the harmonic distortion was not produced by the acoustic system or the middle ear, the stapes vibration was measured in each animal.

### Statistics and reproducibility

Igor Pro (Version 8.04, WaveMetrics, Lake Oswego, OR) was used for data processing and statistical analysis. The frequency responses of the reticular lamina and basilar membrane at fundamental f0 and harmonics 2f0 and 3f0 were presented by the magnitude and phase as a function of frequency (Fig. 2a, b, e, f, and Fig. 3a, b, e, f). The baseline shift of the reticular lamina and basilar membrane vibrations was determined by the filtered time responses using a low-pass filter with a cut-off frequency of 2 kHz. The total harmonic distortion (*THD*) was determined at the best frequency of the measured cochlear location, according to $THD=\frac{\sqrt{{D}_{2f0}^{2}+{D}_{3f0}^{2}}}{{D}_{f0}}\ast 100$%, where *D*_2*f*0_, *D*_3*f*0_, and *D*_*f*0_ are the displacements at frequencies 2f0, 3f0, and f0 in nanometers. The relative magnitude of the harmonics was calculated by dividing the harmonic displacement by the fundamental displacement (Fig. 2c, d, Fig. 3c, d). Accumulated phase lags inside the cochlea were obtained by subtracting the stapes phase from the reticular lamina or basilar membrane phase (Fig. 2e, f, Fig. 3e, f). The stapes harmonic phase was obtained by multiplying the measured f0 phase by two for the second harmonic or three for the third harmonic. The phase difference between the reticular lamina and basilar membrane was calculated by subtracting the reticular lamina phase from the basilar membrane phase (Fig. 4e–g). The group delay $\tau$ was derived from $\tau =-\triangle \varphi /2\pi \triangle f$, where $\tau$ is in seconds, $\triangle \varphi$ in radians, $\triangle {f}$ in hertz, and $\triangle \varphi /\triangle {f}$ was obtained through a linear regression based on the phase data. The paired two-tailed *t*-test was used for analyzing the difference between the reticular lamina and basilar membrane total harmonic distortion and for other two-group comparisons, such as the f0 group delay to 2f0 group delay at one-half of the best frequency (Fig. 4a, b), the reticular lamina group delays to basilar membrane group delays (Fig. 4c). A one-way ANOVA was used to test the differences among the f0, 2f0, and 3f0 group-delays and group delay differences, and post hoc multiple comparisons were conducted using the Tukey test. For all tests, *P* < 0.05 was considered statistically significant.

### Reporting summary

Further information on research design is available in the Nature Research Reporting Summary linked to this article.

## Supplementary information

Supplementary Information

Supplementary Data 1

Supplementary Data 2

Supplementary Data 3

Supplementary Data 4

Description of Supplementary Files

Reporting Summary

## Data Availability

The source data for all the graphs and charts in the main figures are available in supplementary data files. All other data are available from the corresponding author upon reasonable request.
