# Supplementary material for: The origin of mechanical harmonic distortion within the organ of Corti in living gerbil cochleae
Source: Commun Biol. 2021 Aug 25;4:1008. doi: 10.1038/s42003-021-02540-0 (PMC8387486; doi:10.1038/s42003-021-02540-0)
Supplement: Supplementary file 1 — Supplementary Information [file 42003_2021_2540_MOESM1_ESM.pdf]

Supplementary Information

The origin of mechanical harmonic distortion within the organ of Corti in living gerbil cochleae

Wenxuan He[1], Tianying Ren[1]*

[1]Oregon Hearing Research Center, Department of Otolaryngology, Oregon Health & Science University, Portland, OR 97239, USA. *e-mail: rent@ohsu.edu

Supplementary Notes

Based on the Muller's frequency-location function[1]: $CF(pos) = 0.398 * \left(10^{(100-pos)*0.022} - 0.631\right)$, where CF is the characteristic frequency in kHz and pos the percentage of the basilar membrane length, we calculated the 20 kHz best frequency location as the distance from the cochlear base d using the following equation: $d = (100 - log\,(20/0.398 + 0.631)/0.022) * 11.1$, where CF is 20 kHz and the basilar membrane length is 11.1 mm in the adult gerbil (Muller 1996)[1]. Our calculation shows that the 20-kHz place is ~ 2.5 mm from the cochlear base. In order to confirm this, we measured the distance from the cochlear base to the 20-kHz best-frequency place in three gerbil cochleae using a 3D positioning system. The magnitude and phase of the reticular lamina and basilar membrane vibrations were measured as a function of the frequency at different sound levels in the sensitive living cochlea. The best frequency was determined by the frequency with a maximal displacement at 40 dB SPL. The focal location of the object beam was moved toward the base or apex according to the best frequency. When the 20-kHz best-frequency location was found, this location was marked by placing a few ~20-μm diameter gold-coated glass beads or by making a small hole on the osseous spiral lamina using a tungsten electrode. After the animal was euthanized, the left temporal bone was harvested, and the cochlear bony shell was removed. The remaining cochlea with the intact modiolus and the osseous spiral lamina was mounted on a head holder attached to a 3D precision translation stage. When the

object beam was focused on the edge of the osseous spiral lamina at the base, the first set of coordinates x_0, y_0, and z_0 were read out using the position controller. After the focal spot of the object beam was moved toward the apex by ~50 μm along the edge of the osseous spiral lamina, the next set of coordinates x_1, y_1, and z_1 were collected. The distance between the two locations (d) was determined by: $d = ((x_1 - x_0)^2 + (y_1 - y_0)^2 + (z_1 - z_0)^2)^{0.5}$. And then, distances between the following points were measured. The best-frequency location was obtained by: $\sum_{i=1}^{n} d_i = d_1 + d_2 + d_3 + \cdots . + d_n$, where d_1, d_2, and d_3 are the distances between the first and second, second and third, and third and fourth location respectively, and d_n is the distance between the best-frequency location and the immediate basal point. Our measurement indicates that the 20-kHz best-frequency location is ~2.4 mm from the base, which is similar to previous measurements[2, 3]. Since the frequency-location map shifts from the base toward the apex as the cochlea matures[1] and the gerbils used in this study (4-8 week old) is younger than those used by Muller (2-4 month), our measurement of the 20 kHz location (~2.4 mm from the base) is consistent with the calculation based on the Muller's frequency-location map (~2.5 mm). The more basal best-frequency location in young gerbils allows the object beam to access the cochlear partition with an angle closer to 90 degree in this study.

The non-perpendicular angle of the object beam with the cochlear partition was minimized by deflecting the beam using a glass coverslip with a proper head position in the present study. After the round window membrane was removed with a tungsten hook, the animal's head was turned to a position with the round window at the top and the apex at the bottom. This head position decreases the angle between the tangent line at the ~20-kHz location of the cochlear partition and the horizontal plane (Fig. 1a). The thick dotted gray line is a mediolateral view of the cochlear partition of the left cochlea in the basal region. The cochlear partition locations in the space were measured using a 3D positioning system as described above. The 20-kHz location (green dot) is ~2.4 mm from the base. One end of a glass coverslip strip with ~1.5-mm width and 0.17-mm thickness was placed on the

posterolateral bony edge of the round window, and the other end on the tympanic ring and the bony edge of the bulla. It usually took a few minutes for perilymph to fill the space between the glass coverslip and the round window. Since the edge of the tympanic ring is in a higher plane than the round window, an angle is formed between the glass coverslip and the horizontal plane. This angle was adjusted by lifting or lowering the glass coverslip at the tympanic-ring end. The glass coverslip with the perilymph below deflects the object beam toward the cochlear base due to refractive index differences among the air (1.00), glass (1.52), and water (1.33) (red lines in Fig. 1a). This consequently brings the object beam close to the perpendicular direction to the cochlear partition. A white light source through a single-mode optic fiber was brought close to the lateral bony wall of the scala vestibuli. The location and orientation of the optical fiber were adjusted so that the landmarks of the cochlear partition are visible. The image in Fig. 1b shows the edge of the osseous spiral lamina over a >100-μm distance along the longitudinal direction. According to the numerical aperture of the objective lens of 0.28 (Plan Apo 20X, NA 0.28, Mitutoyo, Japan) and central wavelength of the illuminating light of ~550 nm, the image depth or the axial resolution is ~8.5 μm. Thus, the image in Fig. 1b indicates a variation of ~8.5 μm in the optical axils over a >100-μm distance in the longitudinal direction. This indicates a <5-degree angle between the tangent line of the cochlear partition at the ~20 kHz place and the plane perpendicular to the object beam. Given the distance between the basilar membrane and the reticular lamina of ~60 μm[4, 5], the estimated <5-degree angle results in a <6-μm change in the longitudinal direction. The <6-μm distance in the longitudinal direction will result in <1.0-μs delay if the estimated wavelength is >300 μm at the 20-kHz best-frequency place, according to previous measurements[3, 6, 7].

To determine the effect of this potential systematic error on the present results, we reanalyzed the group delay differences between the reticular lamina and basilar membrane harmonics after removing a hypothetic 1-μs group delay from reticular lamina delays. The data used for this analysis

are the same as those for Fig. 4c in the revised manuscript. A paired t-test shows that the reticular lamina 2f0 group delay (with correction, 180±10 μs) (without correction, 181±10 μs) is significantly smaller than that of the basilar membrane (197±12 μs) (with correction, t=3.7684, P=0.0044, n=10) (without correction, t=3.5414, P=0.0063, n=10). Moreover, the reticular lamina 3f0 group delay (with correction, 192±7 μs) (without correction, 193±7 μs) is also smaller than that of the basilar membrane (198±6 μs)(with correction, t=2.8653, P=0.0186, n=10) (without correction, t=2.4376, P=0.0375, n=10). In contrast, the reticular lamina f0 group delay (with correction, 196±11 μs) (without correction, 197±11 μs) is significantly larger than that of the basilar membrane (181±9 μs) (with correction, t=2.9024, p=0.0175, n=10) (without correction, t=3.1097, p=0.0125, n=10). These results show that the potential systematic error does not affect the present results significantly.

Supplementary References

1 M. Muller. The cochlear place-frequency map of the adult and developing Mongolian gerbil. *Hear. Res.* **94**, 148-156 (1996).

2 T. Ren. Longitudinal pattern of basilar membrane vibration in the sensitive cochlea. *Proc. Natl. Acad. Sci. U. S. A.* **99**, 17101-17106 (2002).

3 T. Ren, W. He & P. G. Gillespie. Measurement of cochlear power gain in the sensitive gerbil ear. *Nat. Commun.* **2**, 216 (2011).

4 J. A. Soons, A. J. Ricci, C. R. Steele & S. Puria. Cytoarchitecture of the mouse organ of corti from base to apex, determined using in situ two-photon imaging. *J Assoc Res Otolaryngol* **16**, 47-66 (2015).

5 X. Hu, B. N. Evans & P. Dallos. Direct visualization of organ of corti kinematics in a hemicochlea. *J. Neurophysiol.* **82**, 2798-2807 (1999).

6 T. Ren, W. He & E. Porsov. Localization of the cochlear amplifier in living sensitive ears. *PLoS One* **6**, e20149 (2011).

7 W. He, A. Fridberger, E. Porsov, K. Grosh & T. Ren. Reverse wave propagation in the cochlea. *Proc. Natl. Acad. Sci. U. S. A.* **105**, 2729-2733 (2008).
