# Supplementary material for: The origin of mechanical harmonic distortion within the organ of Corti in living gerbil cochleae
Source: Commun Biol. 2021 Aug 25;4:1008. doi: 10.1038/s42003-021-02540-0 (PMC8387486; doi:10.1038/s42003-021-02540-0)
Supplement: Supplementary file 6 — Description of Supplementary Files [file 42003_2021_2540_MOESM6_ESM.pdf]

Description of Additional Supplementary Files

File name: Supplementary Data 1
Description: Source data for Fig. 1.

File name: Supplementary Data 2
Description: Source data for Fig. 2.

File name: Supplementary Data 3
Description: Source data for Fig. 3.

File name: Supplementary Data 4
Description: Source data for Fig. 4.
