# Supplementary material for: The origin of mechanical harmonic distortion within the organ of Corti in living gerbil cochleae
Source: Commun Biol. 2021 Aug 25;4:1008. doi: 10.1038/s42003-021-02540-0 (PMC8387486; doi:10.1038/s42003-021-02540-0)
Supplement: Supplementary file 7 — Reporting Summary [file 42003_2021_2540_MOESM7_ESM.pdf]

nature research

Corresponding author(s): Tianying Ren

Last updated by author(s): 7/28/2021

Reporting Summary

Nature Research wishes to improve the reproducibility of the work that we publish. This form provides structure for consistency and transparency in reporting. For further information on Nature Research policies, see our Editorial Policies and the Editorial Policy Checklist.

Statistics

For all statistical analyses, confirm that the following items are present in the figure legend, table legend, main text, or Methods section.

n/a	Confirmed	
☐	☒	The exact sample size (*n*) for each experimental group/condition, given as a discrete number and unit of measurement
☐	☒	A statement on whether measurements were taken from distinct samples or whether the same sample was measured repeatedly
☐	☒	The statistical test(s) used AND whether they are one- or two-sided<br>*Only common tests should be described solely by name; describe more complex techniques in the Methods section.*
☒	☐	A description of all covariates tested
☒	☐	A description of any assumptions or corrections, such as tests of normality and adjustment for multiple comparisons
☐	☒	A full description of the statistical parameters including central tendency (e.g. means) or other basic estimates (e.g. regression coefficient) AND variation (e.g. standard deviation) or associated estimates of uncertainty (e.g. confidence intervals)
☐	☒	For null hypothesis testing, the test statistic (e.g. *F*, *t*, *r*) with confidence intervals, effect sizes, degrees of freedom and *P* value noted<br>*Give P values as exact values whenever suitable.*
☒	☐	For Bayesian analysis, information on the choice of priors and Markov chain Monte Carlo settings
☒	☐	For hierarchical and complex designs, identification of the appropriate level for tests and full reporting of outcomes
☒	☐	Estimates of effect sizes (e.g. Cohen's *d*, Pearson's *r*), indicating how they were calculated

Our web collection on statistics for biologists contains articles on many of the points above.

Software and code

Policy information about availability of computer code

Data collection	Computer code for data collection available upon reasonable request.
Data analysis	Igor Pro (Version 8.04, WaveMetrics, Lake Oswego, OR) was used for data analysis.

For manuscripts utilizing custom algorithms or software that are central to the research but not yet described in published literature, software must be made available to editors and reviewers. We strongly encourage code deposition in a community repository (e.g. GitHub). See the Nature Research guidelines for submitting code & software for further information.

Data

Policy information about availability of data

All manuscripts must include a data availability statement. This statement should provide the following information, where applicable:

- Accession codes, unique identifiers, or web links for publicly available datasets
- A list of figures that have associated raw data
- A description of any restrictions on data availability

The data that support the finding of this study are available from the corresponding author upon reasonable request.

Field-specific reporting

Life sciences study design

All studies must disclose on these points even when the disclosure is negative.

Sample size	The grouped data reported in this study were collected from ten sensitive cochleae. This sample size was determined according to previous publications on the cochlear mechanical measurements in the sensitive living cochleae of experimental animals. Moreover, two-tailed t test and ANOVA reveals that the harmonic 2f0 delays are significantly greater than f0 at one-half of the best frequency and that the basilar membrane 2f0 and 3f0 delays are larger than those of the reticular lamina harmonics.
Data exclusions	Results from the eight animals were excluded because of hearing loss at high frequencies, poor signal-to-noise ratio of the data, and/or incomplete data sets.
Replication	The reproducibility of the experimental findings was ensured by collecting data at different stimulus levels and from different cochleae across animals. All data reported in this paper are replicable.
Randomization	The order of the measurements of the reticular lamina vibration and the basilar membrane vibration was random.
Blinding	Blinding is not relevant to this study because the data were collected from the same group of gerbils with normal hearing and the reticular lamina and basilar membrane locations have to be determined for the data collection.

Reporting for specific materials, systems and methods

We require information from authors about some types of materials, experimental systems and methods used in many studies. Here, indicate whether each material, system or method listed is relevant to your study. If you are not sure if a list item applies to your research, read the appropriate section before selecting a response.

Materials & experimental systems

n/a	Involved in the study	
☒	☐	Antibodies
☒	☐	Eukaryotic cell lines
☒	☐	Palaeontology and archaeology
☐	☒	Animals and other organisms
☒	☐	Human research participants
☒	☐	Clinical data
☒	☐	Dual use research of concern

Methods

n/a	Involved in the study	
☒	☐	ChIP-seq
☒	☐	Flow cytometry
☒	☐	MRI-based neuroimaging

Animals and other organisms

Policy information about studies involving animals; ARRIVE guidelines recommended for reporting animal research

Laboratory animals	Eighteen young healthy Mongolian gerbils of both sexes age 4 to 8 weeks (40-80 g) were used in this study.
Wild animals	This study did not involve wild animals.
Field-collected samples	This study did not involve samples collected from the field.
Ethics oversight	The animal use protocol was approved by the Oregon Health & Science University Institutional Animal Care and Use Committee.

Note that full information on the approval of the study protocol must also be provided in the manuscript.
